# Ligamentum flavum analysis in patients with lumbar discus hernia and lumbar spinal stenosis

**DOI:** 10.1038/s41598-023-30928-x

**Published:** 2023-03-07

**Authors:** Vuk Aleksić, Jovana Todorović, Nenad Miladinović, Nemanja Aleksić, Vojislav Bogosavljević, Marko Đurović, Svetlana Kocić, Radmila Aleksić, Miloš Joković

**Affiliations:** 1 Department of Neurosurgery, Clinical Hospital Center Zemun, Belgrade, Serbia; 2 Institute for Social Medicine, Belgrade, Serbia; 3 Faculty of Medicine, University of Belgrade, Belgrade, Serbia; 4 Department of Pathology, Clinical Hospital Center Zemun, Belgrade, Serbia; 5 Clinic for Cardiac Surgery, Clinical Center of Serbia, Belgrade, Serbia; 6 Neurosurgery Clinic, Clinical Center of Serbia, Belgrade, Serbia; 7 Department of Radiology, Clinical Hospital Center Zemun, Belgrade, Serbia

**Keywords:** Neurological manifestations, Pain

## Abstract

The normal ligamentum flavum (LF) is a well-defined elastic structure with specific innervation. Several studies investigated LF in patients with lumbar spinal stenosis (LSS) and used lumbar discus hernia (LDH) patients as control group, only on the presumed thesis that LF in this patients have normal morphology. In patients with LSS thickening of the LF is the main cause of stenosis, which is most often presented with neurogenic claudication, whose pathophysiological mechanism is not completely understood. We conducted observational cohort study of 60 operated patients divided into two groups. The first group of 30 patients underwent micro-discectomy (LSH group), and second group with 30 patients underwent decompression, after which analysis of harvested LF was performed. Patients from the LDH group and LSS group differed significantly in the frequencies of chief complaints, duration of symptoms, physical examination, and specific morphological/radiological parameters. The LF analysis showed that the groups differed significantly in the amount of collagen and elastic fibers, as well as in the histological appearance/architectonics of elastic fibers. Also, groups differ in the presence of LF nerve fibers. Our findings speak in favor of the recently postulated inflammatory theory in the origin of spinal neurogenic claudication’s.

## Introduction

North American Spine society defines discal herniation as localized dislocation of disc material beyond the normal margins of the intervertebral disc space resulting in numbness, pain, or weakness in a dermatomal and myotomal distribution^1^. Most common localization of disc herniation is lumbar spine region, and thus, lumbar disc herniation (LDH) represents a major cause of cost and morbidity. Being one of the most common diagnoses in spine practice, the incidence of symptomatic LDH in the United States of America has been estimated at 1% to 2%^2^. The lifetime prevalence of low back pain is about 80%, with disk disorders being the most common cause. The prevalence of a symptomatic LDH is about 1 to 3%, depending on age and sex. The highest prevalence is in persons 30 to 50 years of age, with a male-to-female ratio of near 2:1. In persons 25 to 55 years of age, about 95% of cases of herniated disc occur at the lower lumbar spine region, most commonly at the L4/L5 level. Disc herniation above this spine level is more common in patients older than 55 years^3,4^. On the other hand, the most common spinal disorder in elderly patients is lumbar spinal stenosis (LSS), which causes neurogenic claudication’s, and sometimes lower back and leg pain^5^. LSS occurs as a result of degenerative changes in the spine, including intervertebral discs bulging or herniation, bony proliferation of the facet (zygapophyseal) joints and formation of osteophytes, as well as ligamentum flavum (LF) thickening. Among these, LF thickening is considered to be a major contributor to the development of LSS^5^.

The majority of studies examining morphological changes in the LF in patients with LSS compared findings with changes in the LF of patients with LDH. However, according to our knowledge, no study has firstly shown clinical, morphological or radiological differences between the two diseases, although essentially both lead to a narrowing of the spinal canal at its anatomical base and for many years LSS was defined as any type of narrowing of the spinal canal caused by bone or by soft tissue. Today LDH is considered as a distinct and separate entity. Different clinical pictures, signs and symptoms, as well as morphological differences favor separation of these entities. It is well known that most common symptoms of LSS are neurogenic claudication’s, while the first symptom of LDH is radicular pain or sciatica^6^. In our study we first showed significant differences between age of patients, clinical signs and symptoms, as well as specific morphological/radiological parameters of LSS as experimental group and LDH as control group, which we believe is a unique aspect in methodology and significant improvement over previous studies.

Although many different theories exist, it is currently unknown which exact pathophysiological mechanism causes neurogenic claudication’s as a response to the compression of spinal nerves. The two main proposed mechanisms agree that neurovascular compression plays a significant role. The neuro-ischemic theory proposes that poor blood supply to the spinal nerve roots results in neurogenic claudication’s. In contrast, the venous stasis theory suggests that a combination of low levels of oxygen and metabolite buildup are responsible due to venous backup at the cauda equina. It is recently postulated that inflammatory changes contribute to LF hypertrophy, or that inflammation by itself causes neurogenic claudication’s and pain^7–9^. In our study histological changes of LF are investigated in two different groups after which proposition for new mechanism of neurogenic claudication’s was obtained.

The aim of our study was to firstly show clinical, and radiological/morphological differences between LSS and LDH in order to make a clear distinction between these two entities and thereby enable a comparison of the LF analysis in these two groups of patients, in which patients with LSS would be the experimental group, and patients with LDH the control group. Based on the specific histological findings of the LF, a new assumption was made about the pathophysiological mechanism of neurogenic claudication.

## Results

A total of 60 patients were included in the study, 30 (50%) with lumbar discus hernia (LDH group) and 30 (50%) with lumbar spinal stenosis (LSS group). The most common level of LDH was L5/S1 (16/30), while L4/L5 level was operated in 12 patients (12/30). Two patients had LDH at the L3/L4 level. In the group with LSS 13 patients had one segment stenosis, while 10 patients had 2 level stenosis, and 7 patients had 3 level stenosis. The LF was harvested from the level of the most pronounced stenosis, which was at L4/L5 level in 15 patients, L3/L4 in 11 patients, and L2/L3 in 3 patients.

Almost two thirds of patients in both groups were male (63.3%). The average age in the group with discus hernia was 45.7 ± 14.7 years and in the group with lumbar stenosis was 63.1 ± 8.0 years, *p* < 0.001. Also, the two investigated groups differed significantly in the values of body mass index (BMI) and the presence of comorbidities. The characteristics of patients included in the study are presented in Table 1.

**Table 1:** Demographic and social characteristics of patients included in the study.

Characteristics	LDH group N (%)	LSS group N (%)	*p* value
Sex			
Male	19 (63.3)	19 (63.3)	
Female	11 (36.7)	11 (36.7)	1.000
Age X ± SD (years)	45.7 ± 14.7	63.1 ± 8.0	0.001
BMI* X ± SD (kg/m^2^)	25.7 ± 2.9	26.9 ± 3.9	0.014
Education			
Primary	6 (20.0)	5 (16.7)	
Secondary	11 (36.7)	17 (56.7)	
College	5 (16.7)	3 (10.0)	
Faculty	8 (26.7)	5 (16.7)	0.463
Sitting at work (hours)			
No sitting	8 (26.7)	8 (26.7)	
< 2	3 (10.0)	9 (30.0)	
2–4	5 (16.7)	3 (10.0)	
4–6	5 (16.7)	3 (10.0)	
> 6	9 (30.0)	7 (23.3)	0.373
Level of job difficulty**			
1	1 (3.3)	2 (6.7)	
2	12 (40.0)	6 (20.0)	
3	13 (43.3)	11 (36.7)	
4	4 (13.3)	10 (33.3)	
5	0 (0)	1 (3.3)	0.194
Smoking			
Yes	19 (63.3)	18 (60.0)	
No	11 (36.7)	12 (40.0)	0.791
Co-morbidities			
Yes	17 (56.7)	28 (93.3)	
No	13 (43.3)	2 (6.7)	0.001

*Body mass index (BMI) is defined as the body mass divided by the square of the body height, and is expressed in units of kg/m^2^.

**Patients independently graded the difficulty of their work from 0 to 5 according to physical effort.

Patients from the LDH group and LSS group differed significantly in the frequencies of chief complaints and clinical findings, other complaints, duration of symptoms and on physical examination. Also, the two groups differed significantly in morphological/radiological parameters. These characteristics of both groups are presented in Tables 2 and 3.

**Table 2:** Clinical sign and symptoms of patients included in the study.

Characteristics	DH N (%)	LSS N (%)	*p* value
Chief complaint			
1 (sciatica/radicular pain)	25 (83.3)	5 (16.7)	
2 (motor weakens)	3 (10.0)	0 (0)	
3 (spinal claudication’s)	2 (6.7)	22 (73.3)	
4 (back pain)	0 (0)	3 (10.0)	0.001
Duration of symptoms X ± SD (days)	77.5 ± 27.5	1061.8 ± 576.1	0.001
Free walking distance X ± SD (in meters)	2.5 ± 6.1	15.4 ± 9.9	0.001
Lazarevic sign			
Positive	30 (100.0)	15 (50.0)	
Negative	0 (0)	15 (50.0)	0.001
Bragard sign			
Positive	26 (86.7)	8 (26.7)	
Negative	4 (13.3)	22 (73.3)	0.001
Paresthesia			
Yes	29 (96.7)	29 (96.7)	
No	1 (3.3)	1 (3.3)	1.000
Reflexes			
0 (normal, symmetric)	12 (40.0)	2 (6.7)	
1 (symmetrically decreased)	1 (3.3)	13 (43.3)	
2 (symmetrically absent)	3 (10.0)	9 (30.0)	
4 (decreased or absent on the left side)	7 (23.3)	2 (6.7)	
5 (decreased or absent on the right side)	7 (23.3)	4 (13.3)	0.001
EQoL score X ± SD*	12.2 ± 1.6	11.3 ± 1.7	0.293

*EQoL score is an instrument which evaluates the generic quality of life developed in Europe and widely used^10^.

**Table 3:** Morphological/radiological parameters of patients included in the study.

Characteristics	DH N (%)	LSS N (%)	*p* value
Scoliosis			
Yes	6 (20.0)	11 (36.7)	
No	24 (80.0)	19 (63.3)	0.152
Presence of Schmorl's nodes			
Yes	10 (33.3)	17 (56.7)	
No	20 (66.7)	13 (43.3)	0.069
Presence of vertebral body hemangioma			
Yes	7 (23.3)	8 (26.7)	
No	23 (76.7)	22 (73.3)	0.766
Spondylolisthesis			
Yes	1 (3.3)	4 (13.3)	
No	29 (96.7)	26 (86.7)	0.161
Scoliosis angle X ± SD	13.1 ± 3.5	19.1 ± 4.8	0.027
Lumbar lordosis angle X ± SD	36.5 ± 6.7	32.1 ± 13.2	0.264
Ligamentous interfacet distance X ± SD (mm)	19.5 ± 5.1	5.7 ± 1.5	0.001
Flavum on the left X ± SD (mm)	3.8 ± 1.7	6.1 ± 2.3	0.001
Flavum on the right X ± SD (mm)	3.4 ± 1.5	5.2 ± 1.6	0.001
LL diameter X ± SD (mm)	9.9 ± 2.7	8.9 ± 4.2	0.001
AP diameter X ± SD (mm)	9.3 ± 3.1	4.6 ± 1.3	0.001
Joint angle X ± SD	57.4 ± 10.9	36.5 ± 11.4	0.001
Dura surface X ± SD (cm^2^)	0.9 ± 0.4	0.5 ± 0.2	0.001

**Figure 1:**
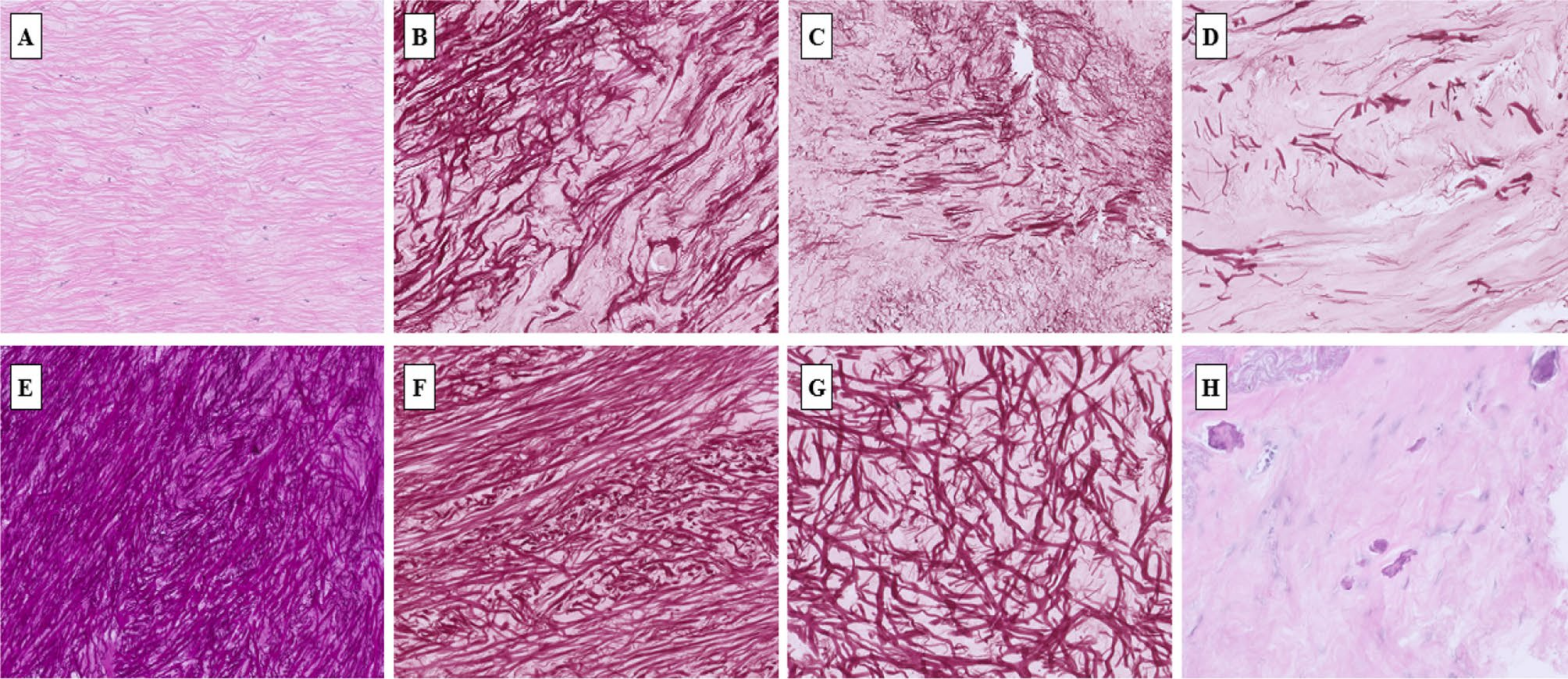
Histological appearance of LF. (**A**) Normal LF with 20% of collagen fibers and 80% elastic fibers (grade 0) and normal architectonics with parallel orientation of more than 90% of elastic fibers (grade 0) (H&E, × 50). (**B**) Mild fibrosis of LF with 20–40% collagen fibers and 80–60% elastic fibers (grade 1) (Orcein, × 50). (**C**) Moderate fibrosis of LF with 40–70% of collagen fibers and 60–30% of elastic fibers (grade 2) (Orcein, × 50). (**D**) Severe fibrosis of LF with over 70% of collagen fibers and less than 30% of elastic fibers (grade 3). (**E**) Moderate disturbance of tissue architectonics with normal (parallel) orientation from 50 to 90% of elastic fibers (grade 1) (Weigert, × 50). (**F**) Moderate disturbance of tissue architectonics with normal (parallel) orientation from 50 to 90% of elastic fibers (grade 1) (Orcein, × 100). (**G**) Severe disturbance of tissue architectonics with normal (parallel) orientation of less than 50% of elastic fibers (Orcein, × 100). (H) Several focuses of calcium depositions indicating grade 1 calcifications (H&E, × 100).

The LF histopathological analysis showed that the groups differed significantly in the amount of collagen and elastic fibers, as well as in the histological appearance/architectonics of elastic fibers (Fig. 1). Immunohistochemicall staining indicated a difference in the presence of nerve fibers between two examined groups. The difference in the presence of macrophages was almost statistically significant, although the value of *p* < 0.05 was not reached. Results of histopathological analysis are presented in the Table 4.

**Table 4:** The LF histopathological analysis.

Characteristics	DH N (%)	LSS N (%)	*p* value
Fibrosis grade			
0	8 (26.7)	0 (0)	
1	17 (56.7)	1 (3.3)	
2	5 (16.7)	11 (36.7)	
3	0 (0)	18 (60.0)	0.001
Calcifications			
Present	9 (30.0)	12 (40.0)	
Absent	21 (70.0)	18 (60.0)	0.417
Architectonics			
Grade 0	12 (40.0)	0 (0)	
Grade 1	17 (56.7)	5 (16.7)	
Grade 2	1 (3.3)	25 (83.3)	0.001
Nerve fibers			
Present	4 (13.3)	11 (36.7)	
Absent	26 (86.7)	19 (63.3)	0.037
Cellularity			
Grade 0 (0 cells/mm^2^)	17 (56.7)	12 (40.0)	
Grade 1 (1–3 cells/mm^2^)	13 (43.3)	13 (43.3)	
Grade 2 (> 3 cells/mm^2^)	0 (0)	5 (16.7)	0.053
Blood vessels			
Present	6 (20.0)	10 (33.3)	
Absent	24 (80.0)	20 (66.7)	0.243

## Discussion

The narrowing of the spinal canal can be caused by degenerative changes such as marked hypertrophy of the facet joints, formation of osteophytes, hypertrophy or thickening of the LF, and disc herniation. However, it is well established that degenerative LSS represents a condition of spinal stenosis usually due to changes in the LF, hypertrophy of the facet joints or due to buckling of the LF secondary to disk height loss, and as such, LSS and LDH are regarded as two different conditions^11^. As the LF covers most of the posterior and lateral part of the lumbar spinal canal, morphological and histological changes merit special attention in the development of LSS. Many studies examined histological changes of the LF in patients with LSS, and in majority of cases control group consisted of the patients with LDH in which it is assumed that the changes in LF are not so pronounced^12,13^. Since degenerative changes in the LF are also increasing with age, especially fibrosis, e.g., the amount of elastic fibers decrease with age and these fibers are replaced by fibrous collagen tissue, compering histological appearance of LF in patients with LDH and LSS can be a methodological pitfall^14–17^. The first part of our study was to show clinical and radiological/morphological differences between groups with LDH and group with LSS, in order to make a clear distinction between these two conditions. There were no significant demographic differences between the two examined groups, e.g., groups did not differ in terms of gender, level of education, job difficulty, sitting time at work nor in smoking cigarettes. On the other hand, groups differ significantly in age, with LSS patients being older. Also, patients with LSS had significantly higher body mass index (BMI), as well as more comorbidities than patients with LDH. Groups differ significantly in clinical findings, e.g., patients with LDH and patients with LSS had significantly different chief complaints, with sciatica/radicular pain being the main complaint in patients with LDH and spinal claudication’s being the most common symptom in patients with LSS. Also, groups differ in the duration of main symptoms, with LSS patients having symptoms in a significantly longer period of time. Differences between examined groups were also significant regarding neurological status e.g., LDH patients more often had a positive Lazarević sign as well as Bragard sign. Also, differences were significant in the type of reflexes, but there were no significant differences of motor strength and presence of paresthesia’s. Finally, groups didn’t show difference in the health-related quality of life determined by the EuroQol EQ-5D questioner, which suggests that the severity of the disease or the impact of the disease on patient’s life is similar between groups. Morphological/radiological investigation showed significant differences of several factors, including scoliosis angle which was more pronounced in the LSS group, as well as ligamentous interfacet distance, laterolateral diameter, and anteroposterior diameter of dural sac, as well as dural surface, which had higher values in patients with LDH comparing to patients with LSS. Interestingly, groups differ significantly in the value of average facet joint angle with higher values in patients with LDH. Most importantly, thickness of the LF on both sides differed significantly between groups, with significantly thicker LF in patients with LSS. According to our study, a clear distinction can be made between the two entities, e.g., LDH and LSS, and thus LDH patients can be a control group for a group of patients with LSS in the study of histological differences in the LF. We believe this is a unique aspect in methodology and significant improvement over previous studies that investigated LF.

According to literature, stenotic changes are most prevalent at the L4/L5 level, followed by the L3/L4 and L5/S1 levels, and about 95% of disc herniation’s occur at L4/L5 or L5/S1^18^. Our findings are in accordance with this data. Groups did not differ from each other regarding gender but differ in age with LDH patients being significantly younger, which is another positive factor in our methodology, since degenerative changes of LF are age related, e.g., LF tends to thicken with increasing age^19^. In our case, another proof that LDH and LSS are two different spine diseases is the significant age difference between the examined groups.

Normal LF is composed of about 80% elastic and 20% collagen fibers. Upon thickening and hypertrophy, LF shows elastic fibers loss and an increase in amount of collagen fibers, resulting in fibrosis. Previous studies indicated that LF hypertrophy was characterized by fibrosis, loss of elastic fibers, increased collagen fibers, calcium depositions, ossification, degeneration of elastic fibers with disturbance of tissue architectonics, and chondrometaplasia^20,21^. In our study there was significant difference between degree of LF fibrotic changes, e.g., in patients with LSS LF showed more pronounced and severe fibrosis with significant loss of elastic fibers and increase in amount of collagen fibers all of which is followed by significantly damaged architecture of the remaining elastic fibers. Described histological changes are similar to scarring during the post-inflammatory repair process in other tissues^21^. Sairyo et al. postulated so called scar-repair-scar theory in which mechanical stress produces damage of LF which is the initial trigger for an inflammatory reaction and subsequent development of tissue scarring^22^. It is also postulated that cells of LF interact with macrophage-like cells to produce angiogenesis-related factors, and that activated LF cells exposed to macrophages can impact the stimulus of angiogenesis-related factors. This implies that fibrosis and scarring, triggered by an inflammatory reaction, is the major mechanism of hypertrophy of LF. So, inflammatory reactions are initiated by trauma such as mechanical stress and are followed by the repair process. Further on, hypertrophied and thickened LF, containing less elastic and more collagen fibers is vulnerable to repetitive flexion–extension motion resulting in a vicious cycle of scar-repair-scar process^21^. Reduced elasticity is certainly contributed by calcification or ossification of the LF, which is also seen as part of pronounced degenerative changes in patients with LSS, especially in increasing age^13^. However, our study did not show difference in calcium deposition between examined groups, probably due to small number of patient included in the study, but majority of other histological features that would indicate this scar-repair-scar process were present in the LF of patients with LSS. In the future studies we are planning to focus on other mentioned LF changes typical for mentioned theory, including chondrometaplasia, mucinous degeneration and ossification, which we believe will be more pronounced in patients in the oldest age group. Lack of LF calcifications in LSS group is probably because our patients were significantly younger than average patients with LSS, e.g. average age of patients in our LSS group was 63.1 years, while majority of patients in others studies are older than 70 years of age^23^.

It is postulated by Chokshi et al. that inflammatory changes surrounding the degenerative facet joints may be the inciting etiology of LF thickening^11^. Also, several studies revealed leakage of inflammatory cytokines from degenerating facet joints, implicating these molecules as causes of both LF thickening and pain generation in the adjacent nerve fibers^24^. It is well established that interleukin-1 (IL-1), a cytokine produced by chondrocytes and other cells in the joint, plays an important role in cartilage degradation by stimulating the synthesis of degradative enzymes that inhibit the production of proteoglycans. Other cytokines that appear to act synergistically with IL-1 to promote matrix breakdown are interleukin-6 (IL-6) and tumor necrosis factor-alpha (TNF-alpha). All of these cytokines are routinely found in inflamed joints^9,25^. In our study an increased presence of inflammation cells, i.e., macrophages in LF were shown in patients with LSS, but statistical significance, although close, was not achieved, probably due to the relatively small number of samples. However, these overall findings suggest chronic inflammation of the LF in patients with LSS.

The major symptom of LSS is neurogenic (spinal) intermittent claudication’s with a decrease in walking distance. Besides claudication’s, low back and leg pain can be seen in patients with LSS^12^. This was also seen in our study with neurogenic (spinal) intermittent claudication’s as the main complaint in patients with LSS.

Although many studies have been performed, clear understanding of the specific pathophysiological mechanism of neurogenic claudication’s remains challenging. The main theories propose that neurovascular compression plays an important role. The neuro-ischemic theory suggests that poor blood and oxygen supply to the spinal nerve roots results in claudication’s. In contrast, the venous stasis theory proposes that a combination of low blood oxygen levels and metabolite buildup are responsible due to venous backup at the nerves of cauda equine. Increasing pain, numbness and gait disturbance with walking may be explained by the corresponding increase in nerve root oxygen requirements^26^. The third and most recent theory implies that chronic inflammation itself leads to the appearance of pain, as a result of the inflammatory response. Several studies confirmed that inflammation and degeneration due to osteoarthritis of facet (zygapophyseal) joint play’s important role in pathogenesis of LSS and neurogenic claudication’s, e.g., a cytokine Interleukin-1 (IL-1) produced by chondrocytes, macrophages and other cells in the joint, plays an important role in cartilage degradation by stimulating the synthesis of degradative enzymes that inhibit the proteoglycans production. All of these molecules i.e. inflammatory cytokines are consistently found in inflamed joints. Chondrocytes are responsible for maintaining the natural balance between degradative enzymes and their inhibitors. In osteoarthritis, there is an imbalance between the levels of destructive and degradative enzymes such as matrix metalloproteinase (MMPs), and their inhibitors. Osteoarthritis affects the articular cartilage and underlying bone, as well as adjacent joint structures. Progressive cartilage degeneration results in narrowing of the joint space which is frequently seen in LSS patients. Areas of bone stripped of cartilage have significant loss of the shock-absorbing mechanism.This causes underlying subchondral bone to form a new articulating surface in the joint and become polished and smooth. In subchondral plane, osteoblasts begin to form new bone tissue and osteophytes in response to messenger molecules secreted by the chondrocytes, which leads to bone remodeling and formation of cartilaginous and bony overgrowths and osteophytes. These changes that occur within the joint are due to inflammatory response and inflammation^24^. New and recent studies have revealed higher production and leakage of inflammatory cytokines from degenerating zygapophyseal joints, implicating these molecules as causes of both LF thickening as well as pain generation in the adjacent nerve fibers^26^. This inflammation theory has its root in the fact that the symptoms of degenerative spine pathology, especially in patients with LSS are often disproportionate to the underlying extent of the disease, e.g., the degree of spinal stenosis does not always correlate with the severity of symptoms and claudication’s^17^. In our study the overall histological appearance of LF in patients with LSS resembles histological appearance similar to tissue scarring during the post-inflammatory repair process in other organs, which is in favor of the inflammatory theory of spinal claudication’s. As mentioned, an increased presence of inflammation cells, i.e., macrophages in LF were shown in patients with LSS, but statistical significance, although close, was not achieved, probably due to the relatively small number of samples. In future studies we will evaluate types of cell proliferation based on immunohistochemistry analyses. Also, recent study of Salimi et al. revealed hyper-expression of biglycan in hypertrophied LF and authors postulated that biglycan may play a crucial role in the pathophysiology of LF hypertrophy through cell proliferation and myofibroblastic differentiation. This speaks in favor of our theory that smothering chronic inflammation could be one of the mechanisms in the origin of spinal neurogenic claudication’s, since it is well established that biglycan initiates and perpetuates the inflammatory response by binding to innate immunity Toll-like receptors (TLR) 2 and 4^27,28^.

In recent years, Banditz et al. studied the phenomenon of nociceptive nerve fiber sprouting, called sensory hyper-innervation which was found in different diseases with inflammatory and non-inflammatory origin such as rheumatoid arthritis, Achilles tendinosis, Crohn's disease, Dupuytren contracture, chronic Charcot foot, and anterior knee pain. They summarize that sensory hyper-innervation is common in many musculoskeletal diseases. They also found a higher density of sensory nerve fibers in LF of LSS patients. These findings support the role of LF in associated low back pain. Density of sensory nerve fibers was positively correlated with typical markers of clinical pain and functional disability. Inflammation as estimated by macrophage infiltration and higher vascularity didn’t play a marked role in LF in their LSS patients^12^. Similary, in the study of Hulmani et al. comparison of LF between patients with LDH and LSS did not show a difference in vascularization and granulation tissue^17^. In our study, patients with LSS showed increased presence of inflammation cells, i.e., macrophages in LF but statistical significance, although close, was not achieved. Same as in study of Banditz et al. and the study of Hulmani et al. we did not find higher vascularity of LF in patients with LSS, but we showed that patients with LSS have more myelinated nerve fibers in LF than patients with LDH. Recently, a sprouting of autonomic sympathetic fibers into the upper dermis of the skin, an area that is normally devoid of them, was found in the skin following chronic inflammation of the rat hindpaw. Longo et al. showed that that transmitters released from the sprouted sympathetic fibers in the synovial membrane and upper dermis contribute to the pain associated with arthritis. Blocking sympathetic fiber sprouting may provide a novel therapeutic approach to alleviate pain^29^. This phenomenon of nociceptive nerve fiber sprouting in LF of patients with LSS can be result of chronic inflammation, since overall histological appearance of LF is similar to tissue scarring during the post-inflammatory repair process in other organs with significant decrease in the number of elastic and increase in the number of collagen fibers with impaired tissue architecture. In our study symptoms in patients with LSS lasted significantly longer than in patients with LDH which also speaks in favor of long-acting chronic smoldering inflammation.

## Conclusions

After proving that LDH and LSS are two different clinical, and radiological/morphological entities, we conducted a histopathological analysis of the flavum in patients of both groups. In our study, it was shown that LF in patients with LSS shows signs of fibrosis and scarring with reduced amount of elastic and an increased amount of collagen fibers and impaired tissue architectonics. Also, significantly more often myelinated nerve fibers were detected in LF of patients with LSS, probably of nociceptive type. This finding speaks in favor of the inflammatory theory in the origin of spinal neurogenic claudication’s, in which the recently described phenomenon of nerve sprouting probably plays a significant role. Defining a definitive theory on the origin of spinal neurogenic has important implications for possible prevention and possibly new and different methods of treatment of this most common degenerative disease of the spine in elderly people. Further studies with many more patients and with specific biochemical analyses are needed to confirm this de novo theory.

## Methods

All procedures and protocols were conducted in accordance with the principles of Helsinki Declaration, written informed consent was obtained from all participants in accordance with standard operative procedures. The study was approved by the Clinical Hospital Center Zemun- Belgrade by the number of approval 8946.

The present study was an observational cohort study of 60 patients who were prospectively recruited and divided into two groups. Patients were hospitalized at the Department of Neurosurgery at Clinical Hospital Center Zemun between 2020 and 2021 and were operated with surgical indications of lumbar disc herniation (LDH) and lumbar spinal stenosis (LSS). All patients fulfilled the following criteria: 1. Age > 18 years, 2. No previous surgery on spine, 3. Diagnosis was verified by magnetic resonance imaging. Patients with history of osteoporosis, immunosuppression, chronic corticosteroid use, intravenous drug use, fever of unknown origin, history of malignancy, unexplained weight loss, or progressive/disabling symptoms were excluded from the study. All patients were operated by one neurosurgeon (V. A.). The LF samples were obtained from the 60 patients randomized in 2 groups. The first group underwent micro-discectomy for LDH and included LF samples from 30 patients (LDH group). The second group underwent decompressive surgery without instrumented fusion for LSS and included LF samples from 30 patients (LSS group). In the patients with multisegmental stenosis, samples were taken from the radiologically determined site of greatest stenosis. While every effort was made to remove the LF en-bloc, in the majority of cases, the LF was removed piecemeal.

**Figure 2:**
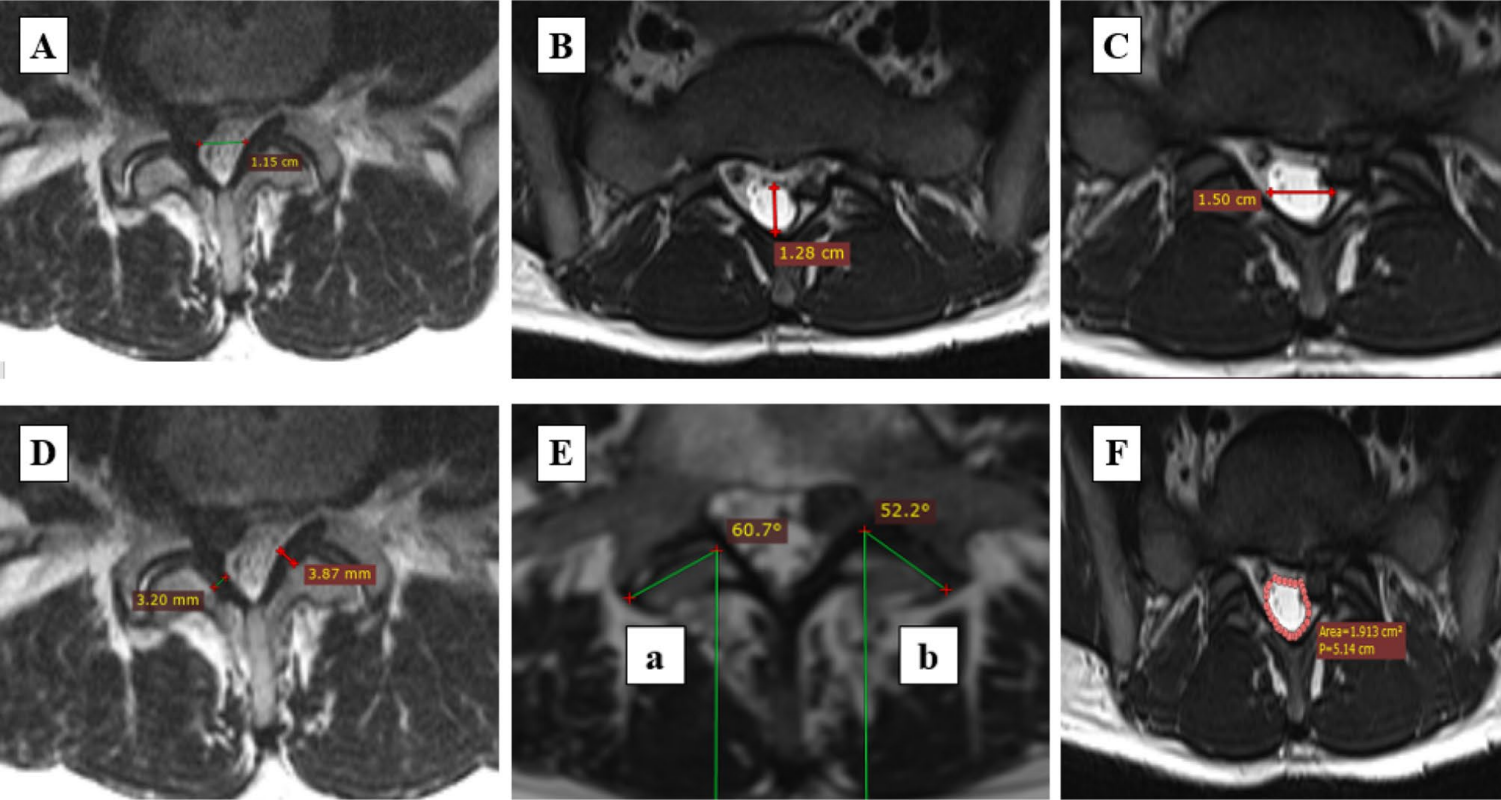
Measurement of (**A**) ligamentous interfacet distance, (**B**) anteroposterior diameter of dural sac, (**C**) laterolateral diameter of dural sac, (**D**) thickness of LF on both sides, (**E**) average facet joint angle measured according to formula: (a + b)/2, (**F**) dural sac surface.

Demographic and clinical data were obtained using a pre-prepared questionnaire as well as data from medical history. Morphological/radiological data were obtained by measuring specific parameters on magnetic resonance imaging—T2 sequences, performed by two experienced radiologists, after several repeated measurements. The examined morphological/radiological parameters measured on the sagittal image projection of the lumbosacral spine region were presence of Schmorl's nodes, vertebral body hemangioma, spondylolisthesis, and value of lumbar lordosis angle. Other measurements were performed at the axial image section where the degree of discal herniation or spinal stenosis were most prominent and included: interfacet distance, thickness of LF on both sides, dural laterolateral (LL) diameter and anteroposterior (AP) diameter of dural sac, average facet joint angle, and dural sac surface. The scoliosis angle was also determined using the standard Cobbs method on the coronary sections of spine magnetic resonance imaging of the patients^30^. Spondylolisthesis was determined as a percentage of vertebral body slippage. Lumbar lordosis angle was also determined using the standard Cobbs method^30^. The determination of the other mentioned parameters is shown in Fig. 2.

### Histopathology processing

The samples were fixed in 10% buffered formalin for 24 h. After fixation, they were dehydrated in a series of ethanol of increasing concentration (30–100%), illuminated in xylene and molded in paraffin. (Histowax, Histowax Product AB, Sweden). Representative tissue sections with a thickness of 3–4 μm were made from paraffin blocks on a rotary microtome (Leica RM2125RT).

After deparaffinization in xylene and rehydration of the sections in a series of decreasing ethanol concentration, the sections were briefly stained with hematoxylin (ICN Biocmedicals Inc., OH, USA), rinsed in tap water and then stained with eosin (Merck, Germany). After washing in water and dehydration in a series of increasing concentrations of ethanol, as well as illumination in xylene, coverslips were mounted with DPX mounting medium (Sigma-Aldrich, Spain).

After deparaffinization and rehydration, the sections were oxidized with a 0.15% KMn04 = 5 solution for 5 min. After washing with tap water, they were decolored with a 2% oxalic acid solution for 10 min. After decolorization, the sections were stained with Orcein ph1 solution for 4 h at room temperature. After staining, the intensity of staining was leveled by incubating the sections in a solution of 1% HCl in 70% alcohol for 30 min. After this step and dehydration in a series of increasing concentrations of ethanol, as well as illumination in xylene, the sections were mounted with DPX medium according to the standard procedure mentioned above.

After fixation, the LF specimens were rinsed several times with 0.1% phosphate buffer to remove the fixative solution and were incubated in 1% osmium tetroxide for 2 h. Then the tissue pieces were dehydrated in ethanol (50%, 70%, 95%, 100%) and treated for 30 min with a 2:1 mixture of propylene and epon. The pieces were embedded in Durcupan. Afterwards, all slices were processed with a dissectional microscope and cut by an ultramicrotome. Ultrathin sections of 60 nm thickness were taken and contrasted with 2.5% uranyl acetate, lead nitrate, and sodium citrate. Hitachi 500 electron microscope was used mainly for detection of nerve fibers.

We used several types of elastic/collagen fibers staining procedures in order to more precisely determine the degree of fibrosis, e.g., the amount of elastic and collagen fibers.

After deparaffinization and rehydration, the sections were stained with commercial sets—Congo red Highman and Weigert rapid method (Bio-Optica, Milano, Italy).

All histopathological slides were studied as part of the examination performed by two experienced pathologists, and the following parameters were noted: collagen/elastic fibers ratio e.g. fibrosis score which was evaluated semi-quantitatively with respect to the entire area of the examined tissue and graded with reference to its severity ranging from 0–3, where value of 0 represented normal finding (20% collagen and 80% elastic fibers), value of 1 represented mild fibrosis (20–40% collagen and 80–60% elastic fibers), value of 2 represented moderate fibrosis (40–70% collagen and 60–30% elastic fibers), and value of 3 represented severe fibrosis (over 70% of collagen and less than 30% of elastic fibers); Tissue architecture was evaluated semi-quantitatively with respect to the entire area of the examined tissue and graded from 0 to 2. Grade 0 indicated normal (parallel) orientation of more than 90% of elastic fibers. Grade 1 indicates moderate disturbance of tissue architectonics with normal (parallel) orientation from 50 to 90% of elastic fibers. Grade 2 indicates severe disturbance of tissue architectonics with normal (parallel) orientation of less than 50% of elastic fibers. Cellularity which depicts inflammation was assessed semi-quantitatively and graded according to number of cells per mm^2^ (grade 0–2). Grade 0 indicated no cells per mm^2^, grade 1 indicated moderate macrophage infiltration with 1 to 3 macrophages per mm^2^. Grade 2 indicated severe macrophage infiltration with more than 3 macrophages per mm^2^. Presence of nerve fibers was implied as a dichotomous variable (no/yes) The same was implied for presence of blood vessels. Calcifications were evaluated semi-quantitatively with respect to the entire area of the examined tissue and grade 1 indicated 0 or 1 calcification focus per mm^2^, and grade 1 indicated 2 or more calcification focuses per mm^2^. All parameters were compared between LDH group and LSS group.

### Statistical analyses

Statistical analyses were done using the methods of descriptive and analytical statistics. The differences between the categorical variables were examined using the Chi-square test and the differences between the numerical variables without the normal distribution were examined using the Mann–Whitney U test. Normality was examined using the Kolmogorov Smirnov and Shapiro Wilk test. The correlations were examined using the Spearman correlation. All statistical analyses were done using the Statistical Package for Social Sciences SPSS 22.0 for Windows. Significance was set at *p* < 0.05.

### Informed consent

Informed consent was obtained from all participants involved in the study.

## Data Availability

Data can be made available upon request from corresponding author VA on E-mail: aleksicvuk@hotmail.com.
